# Mode of Birth and Specific Learning Disabilities: A Systematic Review

**DOI:** 10.7759/cureus.85459

**Published:** 2025-06-06

**Authors:** Maria A Makri, Dimitrios Chaniotis, Victoria V Vivilaki, Effie G Papageorgiou

**Affiliations:** 1 Department of Biomedical Sciences, University of West Attica, Athens, GRC; 2 Department of Midwifery, University of West Attica, Athens, GRC

**Keywords:** cesarean section, child development, learning difficulties, mode of delivery, specific learning disabilities, vaginal birth

## Abstract

In many countries, including Greece, cesarean section (CS) birth rates have exceeded globally accepted levels and the European average, raising concerns about health risks. CS has been linked to adverse outcomes in children, including obesity, metabolic disorders, type 1 diabetes, and neurodevelopmental conditions. Some research suggests lower cognitive performance in CS-born children compared to vaginally born peers. However, the connection between CS and specific learning disabilities (SLDs) remains unexplained and has yet to be clarified. This systematic review aims to assess studies exploring the potential link between CS and SLDs. A systematic search was conducted in Scopus, PubMed, and ScienceDirect from inception to January 2024, following the Preferred Reporting Items for Systematic Reviews and Meta-Analyses guidelines (PROSPERO registration: CRD 42022383362). Relevant keywords were used to identify observational studies, and the Joanna Briggs Institute’s Critical Appraisal Checklist was employed to assess methodological quality. From 1,921 screened titles and abstracts, 219 studies were selected for full-text review, and 10 met the inclusion criteria. Most studies used registry data, with some employing interviews or validated tests. Eight studies suggested CS as a possible risk factor for SLDs, but after adjusting for confounders, only two showed statistically significant results. Some studies lacked adjustments, and two studies highlighted the distinction between elective and emergency CS as a potential confounder. Given the limited number of studies and the variability in their findings, the evidence remains inconsistent in supporting a potential association between CS and SLDs. Future research should adopt a multidisciplinary diagnostic approach and emphasize the use of validated tools for diagnosing SLDs, account for confounding variables, and distinguish between elective and emergency CS procedures.

## Introduction and background

In recent years, growing concerns have emerged regarding the increasing prevalence of cesarean section (CS) deliveries. The World Health Organization (WHO) has also expressed concern about these continuously rising rates [1]. In line with these concerns, global data indicate that CS rates continue to rise persistently in many countries [2,3]. Notably, a recent study reported a median CS rate of 25% across 31 European nations [4]. In Greece, the frequency of CS has risen sharply over the past 40 years, surpassing 50% of all deliveries by 2016 [5]. This excessive use of CS can be attributed to a range of medical and non-medical factors. Non-medically indicated reasons include maternal request due to fear of pain, advanced maternal age, post-term pregnancy, large fetal size, previous CS, in vitro fertilization, and multiple gestations. Medically justified indications involve abnormal fetal position, maternal health conditions, disproportion between fetus and pelvis, fetal macrosomia, umbilical cord complications, amniotic fluid abnormalities, and cases of dystocia or placental/uterine pathology [6]. Consequently, understanding the reasons behind CS deliveries is crucial for interpreting any potential links with other factors, as the risk may vary depending on whether the procedure was medically indicated or not.

Recent studies have suggested a potential correlation between CS and an increased risk of adverse health outcomes in children, including obesity [7,8], atopic dermatitis [9], food allergies [10], respiratory symptoms and disorder [11-13], type 1 diabetes [14], and certain neurodevelopmental and psychiatric conditions [15], as well as attention-deficit/hyperactivity disorder (ADHD) and autism spectrum disorder (ASD) [15,16]. Furthermore, a systematic review indicates that children born via CS tend to exhibit lower cognitive performance in at least one domain compared to their vaginally born counterparts [17]. However, it remains unclear whether CS is associated with the development of specific learning disabilities (SLDs), which are known to involve significant cognitive impairments.

SLDs such as dyslexia, dyspraxia, and dyscalculia are neurodevelopmental disorders that negatively impact school and academic performance by causing difficulties in one or more areas, including learning and comprehension, oral communication, writing and written expression, reading skills, numerical perception and calculation, and mathematical problem-solving [18]. These difficulties are not attributable to language deficiencies, intellectual disabilities, sensory impairments, or other psychiatric, neurological, or psychosocial conditions. Additionally, they cannot be explained by low cognitive functioning, as students with SLDs possess at least an average intelligence quotient [19]. Consequently, SLDs are defined as the inability to meet grade-level expectations in one or more of these areas, despite having access to appropriate learning opportunities tailored to the child’s developmental stage and age [20]. SLDs are distinct from neurodevelopmental conditions such as ASD and ADHD. While all three are classified under neurodevelopmental disorders in both the Diagnostic and Statistical Manual of Mental Disorders (DSM-5) and International Classification of Diseases 11th Revision (ICD-11), they differ in core features, diagnostic criteria, and intervention approaches. SLDs primarily affect specific academic skills (e.g., reading, writing, or math), whereas ASD involves deficits in social communication and restricted behaviors, and ADHD is characterized by inattention, hyperactivity, and impulsivity [18].

SLDs are multifactorial disorders that persist throughout life. It is estimated that 5% to 15% of students across different countries and cultures are diagnosed with SLDs [18]. However, these rates may vary depending on sample size, inclusion criteria, and accepted definitions [21,22]. The origin of SLDs is a topic that has sparked extensive discussion among scientists. Although they have not yet identified all potential causes of SLDs, they have detected numerous risk factors. Some etiological factors are associated with the function of the brain and the neural structure [23,24]. Other factors are related to genetics. According to studies, 30-80% of the observed divergence in reading, math, and spelling performance can be attributed to heritable factors and gene abnormalities [25,26].

In addition, according to recent studies, environmental variables are possible indicators of SLDs. These variables are related to the prenatal, perinatal, and postnatal periods of children. More precisely, a child’s growth and development may occasionally be hampered by specific environmental factors, which might result in SLDs. At the prenatal stage, the diet of the mother, pre-existing medical conditions, the consumption of drugs including alcohol, toxic agents, environmental contaminants, and tobacco smoke may cause adverse effects on the developing fetus [27,28]. During the perinatal period, mode of delivery [29,30], low Apgar score [29], prematurity [31,32], and low birth weight [33-35] are high-risk factors for neurodevelopmental deficits. Postnatal early exposure to general anesthesia may also be linked to SLDs [36]. Finally, maternal [37] and paternal age [38] as well as breech presentation [39] have a significant role in an infant’s subsequent growth. In contrast with all the above, breastfeeding is found to have a positive and large effect on cognitive development [40].

Regarding the mode of delivery, there are differences between CS and vaginal delivery, but the key question is how these differences impact a child’s learning and developmental processes. It is postulated that the initial microbial colonization of the infant gut during birth plays a vital role in subsequent health, cognitive function, neuronal growth, and brain development. Birth mode is a significant factor influencing an infant’s gut microbiome [41]. Consequently, the gut microbiome of infants born via CS differs from that of those born vaginally. More specifically, the gut microbiome of a vaginally delivered infant closely resembles the maternal vaginal and fetal microbiome, as it is shaped by the vertical transfer of the mother’s microbes during passage through the birth canal [42]. In contrast, infants born via CS acquire a gut microbiome composed primarily of horizontally transferred bacteria. These bacteria originate from the mother’s skin and other external sources, including surgical environments, depending on the place of birth [42,43].

Nevertheless, other factors also influence the composition of an infant’s gut microbiome, including the type of CS-elective versus emergency (following labor). Infants who experience labor before an emergency CS have a different microbiome profile compared to those born via elective CS [44]. Beyond delivery mode, other key factors shaping gut microbiome composition include breastfeeding, infant feeding patterns, and antibiotic exposure [45,46]. In general, the gut microbiome plays a crucial role in infant development, as research highlights a bidirectional communication pathway between the gut and brain. This connection influences cognitive, behavioral, and mood-related outcomes through its interaction with the central nervous system [47-49].

Based on the aforementioned factors, it is hypothesized that the mode of delivery may influence a child’s developmental and learning outcomes related to SLDs. Therefore, this review aims to gather and analyze all available data to assess the potential association between mode of birth and the occurrence of SLDs in children.

## Review

Methodology

This systematic review was conducted in keeping with the Preferred Reporting Items for Systematic Reviews and Meta-Analyses (PRISMA) guidelines [50]. This study was registered with PROSPERO (registration ID # CRD42022383362).

Search Strategies

Search terms and synonyms were used for the following key terms: “mode of birth,” “mode of delivery,” “cesarean delivery,” “vaginal delivery,” and “learning disabilities/difficulties/disorders/impairments,” “dyslexia,” “special education,” “special educational needs” and were linked with the Boolean operators OR and AND. Three databases were searched (PubMed, ScienceDirect, Scopus) for observational studies, published in peer-reviewed journals from inception to January 2024. An advanced search was conducted across “all fields” to ensure comprehensive coverage of studies investigating the association between the mode of birth and SLDs, regardless of whether the key terms appeared in the title or abstract. The exact search terms used in this review are provided in the Appendices.

To address conceptual heterogeneity in the terminology and diagnosis of SLDs, consideration was given to a broad range of terms used across the literature to describe conditions corresponding to SLDs. According to the existing literature, various terms, such as “learning difficulties,” “learning disabilities,” or “special educational needs,” are frequently employed to describe what essentially constitutes an SLD. This variation in terminology is both expected and justifiable, given that SLDs encompass a wide spectrum of difficulties (e.g., dyslexia, dyscalculia, dysgraphia), and naming practices differ across diagnostic systems, countries, and educational contexts. To minimize conceptual inconsistency, all studies included used formal diagnostic criteria compatible with DSM or ICD frameworks, or reported multidisciplinary diagnostic procedures. In this review, the term specific learning disabilities will be used to refer to all these difficulties.

Inclusion and Exclusion Criteria

The following inclusion criteria were applied to studies identified through the search: (1) full-text original research published in a peer-reviewed journal, (2) studies reporting any aspect of a potential association between mode of birth and learning disabilities, and (3) articles published in the English language.

We excluded studies that examined (1) anesthesia or analgesia during labor; (2) ASD, ADHD, or intellectual disabilities; (3) developmental delay or emotional/behavioral problems or other impairments; (4) preterm/post-term babies or small/large for gestational age babies or low birth weight babies; (5) babies or children conceived by in vitro fertilization techniques; (6) the impact of specific obstetric or other perinatal complications; (7) babies in breech presentation; and (8) animals, as they are not directly relevant to the research topic or the human population.

Search Procedure and Data Extraction

After compiling the identified studies, duplicates were removed. Titles and abstracts were then screened independently by two researchers to exclude articles with irrelevant content for this systematic review. In the second phase, studies that met the inclusion criteria based on their titles and abstracts, along with those that could not be assessed solely from these sections, were reviewed by a third researcher. Any disagreements between researchers during the screening and review phases were resolved through discussion until consensus was reached. Full-text articles were subsequently assessed by two independent researchers according to the inclusion and exclusion criteria, followed by an eligibility evaluation. Studies deemed eligible were included in this review.

Data extraction was performed following a predetermined strategy. Adjustments for confounders in certain studies, as well as their impact on results, were also recorded. After reviewing the manuscript, all authors provided approval for the final version.

Quality Appraisal and Risk of Bias

The methodological quality and validity of the included studies were assessed using the Joanna Briggs Institute (JBI) Critical Appraisal Checklist [51]. Accordingly, the risk of bias in all studies included in this review was evaluated using JBI tools. The JBI analytical cross-sectional checklist was applied to all studies, as it was deemed appropriate for this review. This checklist consists of eight items designed to assess study quality and risk of bias. Each item is rated as “Yes,” “No,” “Unclear,” or “Not applicable,” leading to an overall assessment of the study. Studies with high risk of bias have positive answers ≤49%; studies with moderate risk of bias have positive answers between 50% and 69%; while studies with low risk of bias have positive answers above 70%.

Results

Results of Search Strategy

The systematic search yielded a total of 2,262 records. After the removal of 341 duplicates, 1,921 unique records were screened based on their titles and abstracts. Of these, 1,690 were excluded for not fulfilling the predefined inclusion criteria. In total, 12 full-text reports could not be retrieved. This unavailability was primarily due to access restrictions (e.g., paywalled articles or lack of an openly accessible version online). In certain cases, attempts were made to contact the corresponding authors to obtain the full text, but no response was received. As these reports could not be adequately assessed for eligibility, they were excluded from the final selection in accordance with the PRISMA 2020 guidelines.

**Figure 1:**
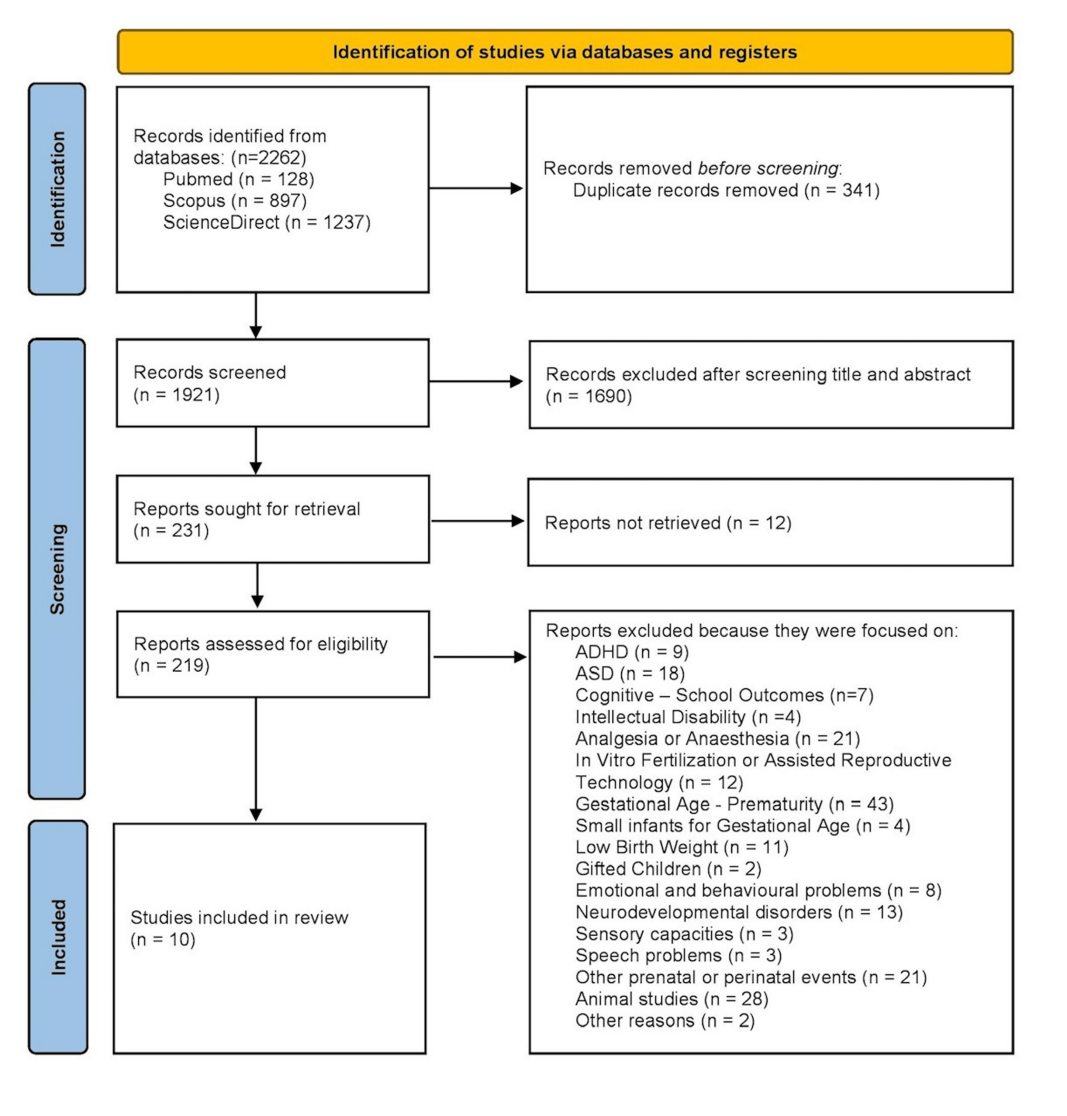
Preferred Reporting Items for Systematic Reviews and Meta-Analyses (PRISMA) 2020 flow diagram.

As a result, 219 full-text articles were assessed for eligibility. During this phase, 209 articles were excluded for reasons such as irrelevant study focus (e.g., outcomes unrelated to the research question, or studies addressing ADHD or ASD) or ineligible populations (e.g., animal studies). Ultimately, 10 studies met the inclusion criteria and were included in the final review. These comprised either prospective or retrospective cohort studies or analyses based on linked data registries. The complete selection process, along with detailed reasons for exclusion, is presented in Figure 1, in accordance with the PRISMA 2020 guidelines.

Several of the included studies utilized intelligence testing alongside other diagnostic tools to collect comprehensive data for the identification of SLDs. This approach is based on a multidisciplinary diagnostic framework, which emphasizes the need to consider multiple domains when evaluating for SLDs. Key components include the individual’s developmental and social history, intelligence quotient (IQ) assessments, standardized academic achievement tests, and psychometric evaluations to support differential diagnosis [18].

Although some studies focused specifically on cognitive performance measures, these were excluded, as low cognitive performance alone is not considered a sufficient indicator of SLDs. Cognitive difficulties typically co-occur with other clinical signs in childhood, given that SLDs are multifactorial conditions affecting a broad range of skills and abilities. Contemporary diagnostic practices rely on specialized assessment tools and multidimensional evaluation methods to ensure diagnostic validity and reliability [18]. Recognizing the importance of a comprehensive, multidisciplinary approach to the diagnosis of SLDs, this review included only those studies that implemented such diagnostic procedures or utilized verified institutional or national datasets.

Characteristics of Included Studies

Study objectives/terminology used: All studies included in this review [52-61] are, in some capacity, related to SLDs, as each of them included elements that point to a potential association between SLDs and mode of delivery. However, four of them [52-54,59] examined neurodevelopmental disorders (NDDs) or special educational needs more broadly but included SLDs in their analysis. The remaining studies used the following terms in their main text: learning disorders, specific learning disabilities, learning difficulties, learning disabilities, dyslexia and developmental dyslexia. Two studies also classified learning disabilities into subtypes, specifying difficulties/disorders/disabilities related to reading, writing, or mathematics/arithmetic [56,60].

The included studies were published between 2001 and 2023. Most were sibling studies, and one was a twin study [60]. Regarding the classification of the mode of delivery, four studies categorized it only as vaginal or CS [52,55,56,60]. Two studies further distinguished between emergency and planned CS [53,54]. One study differentiated vaginal delivery into assisted and unassisted [57], while two others examined vaginal birth after cesarean (VBAC) and repeat CS [58,59]. Additionally, one study classified the mode of delivery into normal, cesarean, and difficult delivery [61] (Table 1).

**Table 1:** Characteristics of the included studies. IQ: intelligence quotient; SLD: specific learning disabilities; CS: cesarean section; DSM-5: Diagnostic and Statistical Manual of Mental Disorders, Fifth Edition; ICD: International Classification of Diseases

Study	Study design, sample size	Participants’ characteristics	Country	Data sources	Methodology/Diagnostic tools/Instruments
Chen et al. (2017) [52]	Prospective cohort study, n = 19,721	Kids born in 2005 according to the National Birth Report Database. Follow-up at 6, 18, and 36 months and 5.5 and 8 years of age	Taiwan	(a) Taiwan Birth Cohort Study (TBCS). (b) National Birth Report Database. (c) Taiwanese Birth Certificates in 2005	(a) TBCS data. (b) Parental/ caregiver interviews. (c) DSM-5 diagnosis by specialists
Chen et al. (2021) [53]	Prospective cohort study, n = 19,142	Kids born in 2005 according to the National Birth Report Database. Follow-up at 6, 18, and 36 months and 5.5 and 8 years of age	Taiwan	(a) Taiwan Birth Cohort Study (TBCS). (b) National Birth Report Database. (c) Taiwanese Birth Certificates in 2005	(a) TBCS data. (b) Parental/ caregiver interviews. (c) DSM-5 diagnosis by specialists
Zhang et al. (2021) [54]	Longitudinal population-based birth cohort study, n = 1,179,341 (6,455 learning disorders)	Term-birth singletons born between January 1, 1990 and December 31, 2003 and followed up from birth until the first diagnosis, emigration, death, or December 31, 2013	Sweden	(a) The Medical Birth Register. (b) The Multi-Generation Register. (c) The National Patient Register (records according to ICD9-ICD10). (d) The Prescribed Drug Register. (e) The Longitudinal Integrated Database for Health Insurance and Labour Studies. (f) The Total Population Register. (g) The Cause of Death Register	Records from the aforementioned registers were linked
Bandla et al. (2017) [55]	Cross-sectional study, n = 96	6–12 years (average IQ >90)	South India	(a) Data from a group of SLD kids in remedial classes (Dyslexia Association of Andhra Pradesh, Hyderabad). (b) Information from two urban and two semi-urban non-government, private, organized English-medium schools	(a) Semi-structured intake proforma. (b) Raven’s and MISIC (IQ). (c) NIMHANS Index (SLD diagnosis). (d) Checklist (for LD identification). (e) DPCL, LD checklist (psychopathology screening)
Görker et al. (2017) [56]	Cross-sectional study, n = 2,174	Students aged between 7 and 13 who attended primary schools in Edirne City Center during the spring of the academic year 2013–2014 and were in the second, third, and fourth grades	Edirne (Turkey)	Data from schools, teachers, parents, and children using specific forms which were completed only by parents and teachers	a) Specific Learning Difficulties Symptom Scale. (b) Learning Disorder Symptom Screening Scale. (c) Sociodemographic data forms
Eaton et al. (2001) [57]	Prospective study, n = 3,420 (580 learning disorder) + 10% from 102,905 reference population	3,420 cases (psychiatric admission <15 years) +10% reference sample, random controls (singletons, 1973–1990 births, survived first year)	Denmark	(a) Danish Psychiatric Case Register - data on admissions to Danish psychiatric institutions (records according to ICD8). (b) Medical Birth Register	Records from the two aforementioned registers were linked
Black et al. (2016) [58]	Record-linkage retrospective cohort study, study population n = 8,218 (from n = 40,145)	Second-born, term, singletons who were delivered between January 1, 1993, and December 31, 2007, who were at least 37 weeks’ gestation from mothers with a history of one prior CS and followed up until January 31, 2015	Scotland, United Kingdom	(a) Scottish Morbidity Record 02 (SMR02). (b) Child Health Systems Programme School. (c) Scottish Morbidity Record 01 (according to ICD10). (d) The Prescribing Information System. (e) Scottish Care Information Diabetes Collaboration. (f) Support Needs System. (g) Scottish Cancer Registry. (h) National Records of Scotland	Records from the all the aforementioned registers were linked
Fitzpatrick et al. (2021) [59]	Population-based record linkage cohort study, n = 44,892 (n = 545,185 before exclusions)	Singleton kids born (at 37–41 completed weeks of gestation) in Scotland between 2002 and 2011 to mothers who had one or more CSs in the past	Scotland, United Kingdom	(a) National Records of Scotland (NRS) live births and stillbirths. (b) The Scottish Morbidity Record Maternity Inpatient and Day Case dataset (SMR02). (c) The Scottish Morbidity Record General/Acute Inpatient and Day case dataset (SMR01). (d) NRS deaths. (e) The Child Health Surveillance Programme Pre-School system (CHSP-PS). (f) Pupil census	Records from the aforementioned registers were linked
González-Valenzuela et al. (2021) [60]	Retrospective cohort epidemiological study, n = 124 children from 62 twin births	Caucasian children born at the Hospital Materno‐Infantil of Málaga (>32 weeks), who were born in 2005 and were in Year 1 of primary education students aged 6 to12 years old	Málaga, Spain	(a) Clinical records held at Hospital Materno Infantil. (b) Children and mothers who gave birth at the Hospital Materno Infantil of Málaga via an appointment. (c) Psychologists who administrated the tests	(1) K‐BIT Kaufman Brief Intelligence Test. (2) Various subtests taken from the psycho‐pedagogical battery Evalúa‐1. (3) Reading accuracy test, phoetic orthography, and visual orthography tests. (4) Maternity and delivery medical records for the moms and their children
Zou et al. (2023) [61]	Case-control study, n = 312 (60 dyslexic children, 252 control group)	Children in grades 2 through 5 with dyslexia and typically developed children recruited at the same schools for the control group students aged 7 to 12 years old	China	(1) Schools, teachers and psychiatrists. (2) Parents or guardians through a self-designed questionnaire on factors influencing dyslexia	(1) Raven’s Standard Progressive Matrices (SPM) test. (2) The Chinese language test. (3) The Dyslexia Checklist for Chinese Children (DCCC). (4) The Pupil Rating Scale-Revised for Learning Disabilities (PRS). (5) Diagnosis from a psychiatrist using Chinese Reading Ability Test (CRAT) and DSM-5

Data sources/diagnostic tools: All of the extracted studies utilized and linked data from registries as well as other national or institutional databases [52-61]. All diagnoses included in these registries and datasets were made by specialists, in accordance with the DSM or ICD criteria. Two studies conducted additional face-to-face interviews with mothers or primary caregivers [52,53], while four others adopted a multidisciplinary approach using validated intelligence tests [55,56,60,61]. Among these four studies, the first combined an intelligence test with a specific learning disability battery and other checklists to confirm the diagnosis of SLDs [55]. The second study employed a specific learning difficulties symptom scale and a learning disorder symptom screening scale, which were completed exclusively by teachers and parents [56]. The third used an intelligence test alongside various subtests for psycho-pedagogical evaluation [60]. The last study linked data from a self-designed questionnaire with the results of an intelligence test, as well as other tests and checklists administered by teachers or psychiatrists to diagnose SLDs [61]. (Table 1)

Study designs: This review included a range of studies examining the potential relationship between mode of delivery and SLDs, employing different study designs and covering various age groups. Three prospective cohort studies [52,53,57] utilized large birth cohorts with follow-up periods extending up to 15 years [57] and 8 years [52,53]. Additionally, two population-based cohort studies [54,59] analyzed national registry data to assess the long-term impact of cesarean delivery, following children up to age 18 and through childhood, respectively, to evaluate their educational needs. Α record-linkage retrospective cohort study conducted in Scotland examined childhood health outcomes up to five years of age [58], while a retrospective cohort study examining twins investigated the association between mode of delivery and SLDs in this specific population [60]. Furthermore, case-control and cross-sectional studies [55,56,61] focused on SLDs and dyslexia, with participants aged 6 to 12 years. Collectively, these studies reported findings on the potential impact of mode of delivery on childhood development, with data spanning from early childhood to late adolescence (Table 1).

Results of Included Studies

Overview of the evidence on mode of birth and SLDs: Regarding the association between the mode of birth and SLDs, the evidence remains inconsistent. One study found that children born via CS had a 20% increased risk of developing NDDs, including SLDs. According to DSM-5, NDDs encompass a range of conditions, including SLDs, developmental delays, ADHD, sensory integration disorder, and ASD [18]. The study suggested that certain factors, such as birth by CS, might influence children’s neurodevelopment. After adjusting for child and parental characteristics, the odds ratio (OR) for cesarean delivery was 1.22 with a 95% confidence interval (CI) of 1.05-1.42. However, after further adjustment for gestational age, the association was attenuated and was no longer statistically significant when this factor was considered (OR = 1.15, 95% CI = 0.98-1.34) [52] (Table 2).

**Table 2:** Results of the included studies. IQ: intelligence quotient; SLD: specific learning disabilities; NDDs: neurodevelopmental disorders; DD: developmental dyslexia; ID: intellectual disability; GA: gestational age; CS: cesarean section; VD: vaginal delivery; VBAC: vaginal birth after cesarean; ADHD: attention-deficit/hyperactivity disorder; ASD: autism spectrum disorder; SEN: special educational needs; ERCS: elective repeat cesarean section; OR: odds ratio; RR: relative risk; HR: hazard ratio; CI: confidence interval

Study	Results	Interpretation – Other results /Effect size	Adjustment for confounders	Results after adjusting for confounders
Chen et al. (2017) [52]	The prevalence of NDDs was significantly different between birth by CS and VD (4.7% vs. 3.8%). Children born by CS show a statistically significant association with neurodevelopmental disorders (learning difficulties, developmental delays, ADHD, sensory integration disorder, autism) (p < 0.05)	CS born children +20% more posibilities for NDDs, increased risk if GA <37 weeks, decreased risk with GA up to 40–42 weeks/Small-moderate effect size	GA, gender, birth order, maternal age, education, income, GDM, parental asthma, history of gestational diabetes mellitus and asthma, gestational weight gain	CS-NDDs association weakens after adjusting for child and parental characteristics (OR = 1.22; 95% CI = 1.05–1.42) and after GA adjustment (OR = 1.15; 95% CI = 0.98–1.34); strongest when CS <38 weeks
Chen et al. (2021) [53]	Different NDDs rates between birth by CS and VD (5.6% vs. 5.0%; OR = 1.15; 95% CI = 1.00–1.32) (p < 0.05), Emergency CS → higher occurrence NDDs than planned CS Emergency CS vs VD-> OR = 1.38 (95% CI = 1.16–1.65) (p < 0.05)	CS born children increased risk for NDDs at age 8 vs VD, emergency CS +38% risk of NDDs vs. VD, mode of delivery and type of CS may influence childhood NDD appearance, GA <37weeks - NDDs association significant (OR = 1.49; 95% CI = 1.20–1.85), GA = 37weeks – NDDs association significant (OR = 1.29; 95% CI = 1.07–1.56)/Small effect size	GA, children characteristics, socioeconomic status, maternal age at birth, maternal diseases at pregnancy	Emergency CS–NDDs association weakens after controlling confounders (OR = 1.27; 95% CI = 1.06–1.53). Emergency CS–NDDs association further weakens after GA adjustment (OR = 1.22; 95% CI, 1.01–1.47)
Zhang et al. (2021) [54]	Children born via planned CD have a greater likelihood to develop any NDD (HR = 1.17; 95% CI = 1.13–1.22). Additionally, planned CD was linked to learning disorders (HR = 1.15; 95% CI = 1.01–1.30)	Planned/intrapartum CD associated with 10–30% higher risk of NDDs, ADHD, ID; planned CD also linked to ASD, communication and learning disorders/Small-moderate effect size	(1) Parental and neonatal characteristics, maternal comorbidities, pregnancy and birth complications. (2) Familial factors (half-sibling and cousin comparisons)	Associations with NDDs, including learning disorders, attenuated after adjustment for familial factors; no evidence of a causal link
Bandla et al. (2017) [55]	CS (SLD special education group 63.4% and SLD schools group 31.3%) is found to be related to SLD in children (p = 0.02)	Prematurity, CS, delayed speech, and family history of SLD were all significantly associated with SLD. A risk factor seems to be CS/Effect size not reported or calculable	-	-
Görker et al. (2017) [56]	Children born via CS have a 5.675 times increased risk of having mathematic disorder (p = 0.009)	CS, delayed walking, and neonatal jaundice are associated with math disorders, with CS-born children being 5.675 times more likely to be affected/Large effect size	Potential confounders were not explicitly stated in this study	-
Eaton et al. (2001) [57]	Children born by CS appeared to have 50% more possibilities to cope with learning disorders (CS RR =1.7 vs VD RR = 1.38)	Learning disorders are linked to low birth weight, small gestational age, and CS, which is especially associated with the less pervasive learning disorders and the “other” disorder category/Moderate effect size	Only adjusted for gender, year of birth/Mode of delivery not controlled	-
Black et al. (2016) [58]	Learning disability was associated with unscheduled repeat CS when compared with VBAC (3.7% vs. 2.3%; OR =1.65; 95% CI = 1.19–2.31). Learning disability risk was 65% higher after unscheduled repeat CS vs. VBAC, with no significant difference for planned CS	Compared to VBAC, unplanned repeat CS was linked to increased learning disability risk, possibly reflecting risks of labor after a previous CS/Moderate effect size	Maternal age, gestation at birth, maternal Carstairs decile, maternal smoking status, birth weight, year of delivery, male infant, and breastfeeding at 6 weeks	The association between learning disability and unscheduled repeat CS persisted after adjustments (aOR =1.64; 95% CI = 1.17–2.29)
Fitzpatrick et al. (2021) [59]	Children born after planned VBAC had a similar risk of learning disability, dyslexia, or other learning difficulties compared to those born by ERCS (all ORs ~1.02, non-significant). No significant associations were found between mode of birth (ERCS or VBAC, with or without induction) and learning outcomes	Limited evidence on the association between planned mode of birth after previous CS and SENs. No significant difference in learning disabilities between planned VBAC and ERCS/Small effect size	Base model - year of birth, Model A - year of birth, sociodemographic and maternal medical and pregnancy- related factors. Model B - variables in model A and infant-related factors. Model C - variables in model B and for any breastfeeding at 6–8 weeks	Slightly increased risk of SENs (RR = 1.06, 95% CI = 1.01–1.12), other specific or moderate learning difficulties (OR = 1.10, 95% CI = 1.00–1.20), and speech/language disorders (OR = 1.14, 95% CI = 1.00–1.30) with actual VBAC vs. ERCS (fully adjusted models)
González- Valenzuela et al. (2021) [60]	Higher odds of phonetic orthography (42.5% vs. 17.9%; OR = 3.40; 95% CI = 1.47–7.87), visual orthography (40.0% vs. 17.9%; OR = 3.06; 95% CI = 1.32–7.13), and reading accuracy difficulties (42.5% vs. 16.7%; OR = 3.69; 95% CI = 1.58–8.64) in CS vs VD	The mean values for children born vaginally show noticeably higher scores in the learning variables. CS is a potential independent risk factor for problems with phonetic and visual orthography, as well as reading accuracy/Small-moderate effect size	Gestational, obstetric, and neonatal variables (maternal age at delivery, gestational age, fetal presentation, Apgar 1, and newborn weight)	Fetal presentation acted as a potential confounder in the association between delivery mode and phonetic orthography (aOR = 2.50; 95% CI = 1.01–6.18); other results remained unchanged
Zou et al. (2023) [61]	No statistically significant differences were found between DD and delivery mode (p = 0.147)	DD may be influenced by the home reading environment, and various educational, sociometric, and demographic factors/Moderate-to-large effect size	Adjustments for confounders only in the cases of associations between Chinese Dyslexia and the Home Reading Environment/Mode of delivery not controlled	-

Another study reported differences in the prevalence of NDDs between cesarean and vaginal births (5.6% vs. 5.0%, respectively). Children born via emergency CS had a significantly higher incidence of NDDs, including SLDs, compared to those born vaginally (OR = 1.38, 95% CI = 1.16-1.65; p = 0.05). Additionally, the risk of neurodevelopmental problems in the emergency CS group was 38% higher than in children born vaginally. However, after adjusting for covariates (gestational age, children’s characteristics, socioeconomic status, maternal age at birth, maternal diseases during pregnancy), this association weakened [53] (Table 2).

A similar finding was reported in another study, where planned (rather than emergency) cesarean delivery was significantly associated with an increased risk of NDDs (HR = 1.17, 95% CI = 1.13-1.22) and learning disorders (HR = 1.15, 95% CI = 1.01-1.30). While children born via planned cesarean appeared to have a higher likelihood of SLDs, these associations weakened to the null after adjusting for familial factors [54] (Table 2).

Three studies identified a significant correlation between CS and SLDs; however, these studies either did not adjust for confounders [55,56] or accounted for only minimal and insufficient confounders [57]. Specifically, one study found that CS was associated with SLDs in children, with 63.4% of the SLD special education group and 31.3% of the SLD school group being born via CS (p = 0.02) [55]. Another study reported that CS increased the risk of learning disorders by approximately 50% and the risk of “other” disorders by 25%. More precisely, CS was associated with a 70% increased risk (RR = 1.7), while vaginal delivery was associated with a 38% increased risk (RR = 1.38). When directly comparing CS to vaginal delivery, the relative risk is approximately 23% higher for children born via CS [57]. Additionally, one of the above studies found that children born via CS were 5.675 times more likely to experience math difficulties compared to children born vaginally [56] (Table 2).

Two studies examined VBAC and repeat CS, meaning that the study population was singleton children born to mothers who had one or more previous CSs [58,59]. In other words, these studies included mothers who had delivered their first child via CS and later gave birth to their second child either through VBAC or a second repeat CS. One study found that SLDs were more common following unscheduled repeat CS compared to VBAC (3.7% vs. 2.3%; aOR = 1.64, 95% CI = 1.17-2.29). However, although this suggests a higher risk of SLDs following unscheduled repeat CS, the association may reflect complications occurring during labor [58]. In contrast, another study reported that actual VBAC, as opposed to elective repeat CS, was associated with a slight increase in the likelihood of special educational needs (RR = 1.06, 95% CI = 1.01-1.12) and other specific or moderate learning disabilities (OR = 1.10, 95% CI = 1.00-1.20) [59]. Consequently, the second study did not find a strong association between elective repeat CS and SLDs [59]. Both studies accounted for potential confounding factors, thereby increasing the internal validity and partial reliability of their findings (Table 2).

A twin study suggested that CS may be an independent risk factor for SLDs. Children born via CS exhibited a threefold increased risk of difficulties in phonetic (42.5% vs. 17.9%; OR = 3.40, 95% CI = 1.47-7.87) and visual (40% vs. 17.9%; OR = 3.06, 95% CI = 1.32-7.13) orthography, as well as reading accuracy (42.5% vs. 16.7%; OR = 3.69, 95% CI = 1.58-8.64), compared to those born via vaginal delivery, based on the observed statistical association [60]. In this study, all mean values indicated significantly higher scores in learning-related variables for children born via vaginal delivery compared to those born via CS, with a moderate effect size. The results remained consistent across all analyses and adjustments. The only potential confounding factor that altered the relationship between mode of birth and phonetic orthography was fetal presentation (42.5% vs. 17.9%; aOR = 2.50, 95% CI = 1.01-6.18) [60] (Table 2).

Finally, one study found no significant association (p = 0.147) between CS and developmental dyslexia (DD). This study categorized the mode of delivery into vaginal delivery, CS, and difficult delivery, but no correlation was observed. Instead, the study identified various associations between DD and various environmental and social factors [61].

To sum up, eight out of ten studies [52-58,60] found evidence that cesarean was associated with an increased risk of SLDs in the child having not adjusted for any potential confounding factors. However, in three of these studies, the association was attenuated to the null after adjustment for confounders [52-54], whereas in two studies, the association remained significant despite adjustment for potential confounding factors [58,60]. The remaining three studies either did not adjust for confounders [55,56] or included only minimal and insufficient adjustment [57].

Summary of Statistical Analyses and Effect Sizes

All of the aforementioned studies employed statistical analyses of varying robustness. In addition to descriptive data and commonly used techniques such as chi-square tests, parametric and non-parametric analyses, and t-tests, more advanced methods were also applied. Specifically, several studies conducted logistic regression analyses [52,53,56,58,59,60], while two employed the Cochran-Armitage trend test [52,53]. Cox proportional hazards regression was also used [54,58], and other approaches included analysis of variance [55], relative risk estimation [57], Poisson regression [59], and multivariate logistic regression along with LASSO regression modeling [61]. Although effect sizes were not explicitly reported in all the included studies, they were inferred based on numerical outcomes and results. The estimated effect sizes ranged from small [53,59] to small-to-moderate [52,54,60], moderate [57,58], moderate-to-large [61], and large [56]. In one study, effect size estimation was not possible due to insufficient data [55]. Given the variation in effect sizes, it is reasonable to assume that the risk of bias differs among the studies. This variability was assessed using the JBI Critical Appraisal Checklist [51]. The statistical analyses are provided in the Appendices. 

Results of Quality Assessment

As previously mentioned, the methodological quality and validity of the included studies were assessed using the JBI Critical Appraisal Checklist [51]. All studies, regardless of their type, were evaluated using the JBI analytical cross-sectional checklist, as it was deemed appropriate for this review. Table 3 presents the methodological quality of each included study. According to the results, the majority of studies were classified as having a low risk of bias [52-54,58-61], while only three studies were rated as having a moderate risk of bias [55-57]. Based on the classification defined by the authors, the overall risk of bias in this review can be considered low.

**Table 3:** Results of methodological quality assessment through the Joanna Briggs Institute (JBI) quality appraisal tool.

JBI items/Studies	[52]	[53]	[54]	[55]	[56]	[57]	[58]	[59]	[60]	[61]
Were the criteria for inclusion in the sample clearly defined?	Yes	Yes	Yes	Yes	Yes	Yes	Yes	Yes	Yes	Yes
Were the study subjects and the setting described in detail?	Unclear	Unclear	Yes	Unclear	Yes	Yes	Yes	Yes	Yes	Yes
Was the exposure measured in a valid and reliable way?	Yes	Yes	Yes	Yes	Yes	Yes	Yes	Yes	Yes	Yes
Were objective, standard criteria used for measurement of the condition?	Unclear	Unclear	Unclear	Yes	Unclear	Unclear	Unclear	Unclear	Yes	Unclear
Were all confounding factors identified?	Yes	Yes	Yes	No	No	Unclear	Yes	Yes	Yes	No
Were strategies to deal with confounding factors stated?	Yes	Yes	Yes	No	No	Yes	Yes	Yes	Yes	Yes
Were the outcomes measured in a valid and reliable way?	Yes	Yes	Yes	Yes	Yes	Yes	Yes	Yes	Yes	Yes
Was appropriate statistical analysis used?	Yes	Yes	Yes	No	Yes	No	Yes	Yes	Yes	Yes
% Yes	75%	75%	87.5%	50%	62.5%	62.5%	87.5%	87.5%	100%	75%
Risk	Low	Low	Low	Moderate	Moderate	Moderate	Low	Low	Low	Low

Discussion

Overview of the Review Findings

This review aimed to compile existing literature on the potential association between mode of delivery and SLDs. Given the substantial heterogeneity in study designs, outcome definitions, diagnostic tools, and confounder adjustments across the included studies, the authors considered that conducting a meta-analysis would not provide meaningful or reliable summary estimates. Therefore, a narrative synthesis was preferred, and this rationale has now been explicitly stated in this review. 

To our knowledge, this is the first systematic attempt to synthesize findings on this relationship, accompanied by a critical examination of key methodological gaps and inconsistencies in existing research. Although the link between mode of delivery and cognitive/educational outcomes has been explored by other researchers [17], and considering that cognitive performance alone is not a sufficient predictor of SLDs [18], this systematic review specifically examines the potential relationship between mode of birth and SLDs. However, the findings are inconsistent and highly variable. Furthermore, the absence of sufficiently high-quality studies across large and heterogeneous populations limits the ability to draw valid, reliable, and generalizable conclusions.

In general, the findings of this review appear to be inconsistent. Although eight out of ten studies [52-58,60] reported a significant association between CS and SLDs, the observed associations in three of these studies attenuated to the null after adjustment for confounders [52-54]. However, the fact that two studies did not adjust for confounders [55,56] and one study accounted for only minimal and insufficient factors [57] increases the risk of bias. As a result, only two studies [58,60] found a significant association, but this should be interpreted with caution given the complex nature of this relationship. The variability in study designs, sample sizes, and confounding adjustments further complicates the interpretation of results. Interestingly, only two studies [59,61] reported no association between CS and SLDs.

Scope and Diagnostic Approaches of Included Studies

An important finding that emerged from this review relates to the general purpose of the included studies. Six studies [52-54,57-59] examined broader topics, but all identified elements suggesting a notable potential correlation between mode of birth and SLDs. More specifically, three studies focused on NDDs [52-54], one study investigated psychopathology and mental disorders [57], another explored adverse childhood outcomes based on mode of birth [58], and one study examined special educational needs [59].

In contrast, only four studies specifically focused on learning disabilities and their diagnosis using appropriate tools [55,56,60,61], with two of them further analyzing SLDs by subtype. One study focused on difficulties in reading accuracy, phonetic orthography, and visual orthography [60], while another examined impairments in reading, writing, and mathematics [56]. This highlights a major gap in the literature, as few studies have exclusively examined SLDs concerning mode of delivery using standardized assessment criteria. Most of the included studies [52-54,57-59] relied on data from registers. Although the recorded diagnoses were presumably based on internationally accepted criteria such as those of the DSM and ICD, the specific assessment tools used by clinicians, educators, or researchers in each case were not reported. This lack of information about the exact diagnostic instruments may affect the interpretability and comparability of the findings, potentially contributing to inconsistencies.

Elective Versus Emergency Cesarean: Biological Mechanisms and Potential Causal Pathways

Another important finding concerns the type of CS, rather than just the mode of delivery. Some studies distinguished between elective and emergency CS [53,54], while others did not, which may introduce bias in the latter because the type of CS could be a significant confounder influencing the results. Research suggests that children born via elective CS have an increased risk of NDDs compared to those born via emergency CS [54]. Conversely, another study found that emergency CS increased the risk of NDDs compared to children born vaginally [53]. These findings indicate that the timing and medical indications for CS may play a crucial role in neurodevelopmental and educational outcomes; however, the precise mechanisms remain unclear.

One possible explanation is the differences in gut microbial colonization between CS-born and vaginally born children, which can alter immune system maturation [42,43]. Simultaneously, the physiological stress associated with labor during an attempted vaginal delivery, which may precede an emergency cesarean section may induce glucocorticoid release that supports neural circuit development [62]. In contrast, CS may bypass these processes, potentially contributing to altered neuroimmune responses and an increased risk of neurodevelopmental vulnerabilities [63]. Therefore, before considering the various types of mode of birth in future studies, it is essential to clarify the possible causal differences between vaginal birth and CS, as well as among the different types of CS. Moreover, it is important for future research to also consider the underlying indications for CS, such as fetal distress or intrauterine growth restriction, as these factors may independently influence neurodevelopmental outcomes and help disentangle the role of medical necessity from that of delivery mode itself.

Strengths and limitations

A strong point of our review lies in the comprehensive and systematic approach to the literature search, which included a broad range of research articles to identify all available data on the potential correlation between mode of delivery and SLDs. This approach ensured that relevant studies were considered, regardless of whether key terms appeared in the title or abstract, thereby minimizing selection bias.

However, the main limitation of this review is that many of the included studies relied on data collected through questionnaires or registry-based records, which may be influenced by subjective factors [52-54,57-59]. In these cases, it remains unclear how the diagnoses were established, raising concerns about the accuracy and consistency of reported SLDs. Additionally, the small sample sizes in some studies [55,60,61] limit the statistical power and generalization of the findings.

Furthermore, due to the multifactorial and complex nature of SLDs, none of the reviewed studies could establish a causal relationship between mode of delivery and SLDs. Future research should adopt more robust methodologies, including objective diagnostic tools, to better understand this association.

Future directions

SLDs pose significant challenges to a child’s physical and mental well-being and are recognized as multifactorial conditions, influenced by both genetic predispositions and environmental exposures. A comprehensive approach to diagnosis and intervention is therefore essential, integrating medical history, the developmental environment, including social and linguistic background, and interdisciplinary assessment. Future studies should place particular attention on prenatal, perinatal, and postnatal factors, as these play a critical role in shaping neurodevelopment and may influence the occurrence of SLDs. In this review, mode of birth emerged as a potential factor warranting further investigation. Based on these findings, future research should aim to clarify the possible association between mode of delivery and SLDs by collecting detailed data on biological, medical, and developmental variables, including parental health, perinatal conditions, and genetic background. Importantly, distinguishing between elective and emergency CS, as well as accounting for their underlying medical indications, may offer greater insight into how different delivery pathways relate to child development. The use of standardized and validated diagnostic tools specifically targeting SLDs is also crucial for ensuring the reliability of future findings. 

## Conclusions

In conclusion, eight out of ten studies suggested a potential association between CS and SLDs. However, three studies dispute this link, attributing the observed differences between children born via CS and those born vaginally to confounding variables rather than the mode of delivery itself. Additionally, another three studies reported only unadjusted, weak, and inconsistent associations, which should be interpreted with caution given the complexity of the issue. Two studies found a significant association after adjusting for confounders; however, these findings should also be approached cautiously due to the multifactorial nature of the condition under investigation. The variability in study designs, diagnostic criteria, and the extent of confounder adjustment highlights the need for further high-quality research to clarify these associations. To our knowledge, this is the first systematic review to comprehensively synthesize existing literature on the potential association between mode of delivery and SLDs. However, the overall evidence remains inconclusive. SLDs are multifactorial neurodevelopmental disorders with diverse etiologies, and it is crucial to account for a wide range of potential confounders to accurately assess the impact of mode of delivery on these outcomes. Due to the limited and inconsistent data available on this topic, future research is essential to replicate findings across different populations and determine the potential relationship between mode of birth and SLDs.

## Appendices

**Table 4:** Advanced search strategy.

Advanced search in PubMed/ScienceDirect/Scopus
(“caesarean section” OR “caesarean delivery” OR “mode of birth” OR “mode of delivery”) AND (“learning disabilities” OR “learning disability” OR “learning difficulty” OR “learning difficulties”)
(“caesarean section” OR “caesarean delivery” OR “mode of birth” OR “mode of delivery”) AND (“learning deficit” OR “learning disorder” OR “learning impairment”)
(“caesarean section” OR “caesarean delivery” OR “mode of birth” OR “mode of delivery”) AND (“special education” OR “special education needs” OR “special educational needs”)
(“caesarean section” OR “caesarean delivery” OR “mode of birth” OR “mode of delivery”) AND (“dyslexia” OR “dysgraphia” OR “dyscalculia”)

**Table 5:** Characteristics of the included studies. IQ: intelligence quotient; SLD: specific learning disabilities; CS: cesarean section; DSM-5: Diagnostic and Statistical Manual of Mental Disorders, Fifth Edition; ICD: International Classification of Diseases

Study	Study design, sample size	Participants’ characteristics	Country	Data sources	Methodology/Diagnostic tools/Instruments
Chen et al. (2017) [52]	Prospective cohort study, n = 19,721	Kids born in 2005 according to the National Birth Report Database. Follow-up at 6, 18, and 36 months and 5.5 and 8 years of age	Taiwan	(a) Taiwan Birth Cohort Study (TBCS). (b) National Birth Report Database. (c) Taiwanese Birth Certificates in 2005	(1) Assessments and basic characteristics from prospective longitudinal database of the Taiwan Birth Cohort Study (TBCS). (2) mother or primary caregiver in-person survey interviews (parents’ health and well-being, family characteristics, socioeconomic circumstances, child’s development, behavior, and health outcomes). (3) Diagnosis from pediatric psychiatrists and clinical psychologists according to DSM-5
Chen et al. (2021) [53]	Prospective cohort study, n = 19,142	Kids born in 2005 according to the National Birth Report Database. Follow-up at 6, 18, and 36 months and 5.5 and 8 years of age	Taiwan	(a) Taiwan Birth Cohort Study (TBCS). (b) National Birth Report Database. (c) Taiwanese Birth Certificates in 2005	(1) Assessments and basic characteristics from prospective longitudinal database of the Taiwan Birth Cohort Study (TBCS). (2) mother or primary caregiver in-person survey interviews (parents’ health and well-being, family characteristics, socioeconomic circumstances, child’s development, behavior, and health outcomes). (3) Diagnosis from pediatric psychiatrists and clinical psychologists according to DSM-5
Zhang et al. (2021) [54]	Longitudinal population-based birth cohort study, n = 1,179,341 (6,455 learning disorders)	Term-birth singletons born between January 1, 1990 and December 31, 2003 and followed up from birth until the first diagnosis, emigration, death, or December 31, 2013	Sweden	a) The Medical Birth Register. (b) The Multi-Generation Register. (c) The National Patient Register (records according to ICD9-ICD10). (d) The Prescribed Drug Register. (e) The Longitudinal Integrated Database for Health Insurance and Labour Studies. (f) The Total Population Register. (g) The Cause of Death Register	Records from the aforementioned registers were linked
Bandla et al. (2017) [55]	Cross-sectional study, n = 96	6–12 years (average IQ >90)	South India	(a) Data from a group of SLD kids in remedial classes (Dyslexia Association of Andhra Pradesh, Hyderabad). (b) Information from two urban and two semi-urban non-government, private, organized English-medium schools	(1) Semi‑structured intake proforma. (2) Intelligence tests: a. Raven’s progressive matrices. Standard progressive matrices or Colored progressive matrices. Progressive matrices are used as a screening instrument to rule out children with low IQ. b. MISIC: Malin’s intelligence scale for Indian children, an Indian adaptation of Wechsler intelligence scale for children. (3) The NIMHANS (for the diagnosis of SLD). (4) Checklist to identify learning disabilities. (5) DPCL for children (developmental psychopathology checklist)
Görker et al. (2017) [56]	Cross-sectional study, n = 2,174	Students aged between 7 and 13 who attended primary schools in Edirne City Center during the spring of the academic year 2013–2014 and were in the second, third, and fourth grades	Edirne (Turkey)	Data from schools, teachers, parents, and children using specific forms which were completed only by parents and teachers	(1) Specific Learning Difficulties Symptom Scale. (2) Learning Disorder Symptom Screening Scale. (3) Sociodemographic data forms
Eaton et al. (2001) [57]	Prospective study, n = 3,420 (580 learning disorder) + 10% from 102,905 reference population	3,420 cases (psychiatric admission <15 years) +10% reference sample, random controls (singletons, 1973–1990 births, survived first year)	Denmark	(a) Danish Psychiatric Case Register - data on admissions to Danish psychiatric institutions (records according to ICD8). (b) Medical Birth Register	Records from the two aforementioned registers were linked
Black et al. (2016) [58]	Record-linkage retrospective cohort study, study population n = 8,218 (from n = 40,145)	Second-born, term, singletons who were delivered between January 1, 1993, and December 31, 2007, who were at least 37 weeks’ gestation from mothers with a history of one prior CS and followed up until January 31, 2015	Scotland, United Kingdom	(a) Scottish Morbidity Record 02 (SMR02). (b) Child Health Systems Programme School. (c) Scottish Morbidity Record 01 (according to ICD10). (d) The Prescribing Information System. (e) Scottish Care Information Diabetes Collaboration. (f) Support Needs System. (g) Scottish Cancer Registry. (h) National Records of Scotland	Records from the all the aforementioned registers were linked
Fitzpatrick et al. (2021) [59]	Population-based record linkage cohort study, n = 44,892 (n = 545,185 before exclusions)	Singleton kids born (at 37–41 completed weeks of gestation) in Scotland between 2002 and 2011 to mothers who had one or more CSs in the past	Scotland, United Kingdom	(a) National Records of Scotland (NRS) live births and stillbirths. (b) The Scottish Morbidity Record Maternity Inpatient and Day Case dataset (SMR02). (c) The Scottish Morbidity Record General/Acute Inpatient and Day case dataset (SMR01). (d) NRS deaths. (e) The Child Health Surveillance Programme Pre-School system (CHSP-PS). (f) Pupil census	Records from the aforementioned registers were linked
González-Valenzuela et al. (2021) [60]	Retrospective cohort epidemiological study, n = 124 children from 62 twin births	Caucasian children born at the Hospital Materno‐Infantil of Málaga (>32 weeks), who were born in 2005 and were in Year 1 of primary education students aged 6 to12 years old	Málaga, Spain	Data from (a) clinical records held at Hospital Materno Infantil. (b) Children and mothers who gave birth at the Hospital Materno Infantil of Málaga via an appointment. (c) Psychologists who administrated the tests	(1) K‐BIT Kaufman Brief Intelligence Test. (2) Various subtests taken from the psycho‐pedagogical battery Evalúa‐1. (3) Reading accuracy test, phoetic orthography, and visual orthography tests. (4) Maternity and delivery medical records for the moms and their children
Zou et al. (2023) [61]	Case-control study, n = 312 (60 dyslexic children, 252 control group)	Children in grades 2 through 5 with dyslexia and typically developed children recruited at the same schools for the control group students aged 7 to 12 years old	China	Data from (1) schools, teachers and psychiatrists. (2) Parents or guardians through a self-designed questionnaire on factors influencing dyslexia	1) Raven’s Standard Progressive Matrices (SPM) test. (2) The Chinese language test. (3) The Dyslexia Checklist for Chinese Children (DCCC). (4) The Pupil Rating Scale-Revised for Learning Disabilities (PRS). (5) Diagnosis from a psychiatrist using Chinese Reading Ability Test (CRAT) and DSM-5

**Table 6:** Results of the included studies. IQ: intelligence quotient; SLD: specific learning disabilities; NDDs: neurodevelopmental disorders; DD: developmental dyslexia; ID: intellectual disability; GA: gestational age; CS: cesarean section; VD: vaginal delivery; VBAC: vaginal birth after cesarean; ADHD: attention deficit and hyperactivity disorder; ASD: autism spectrum disorder; SEN: special educational needs; ERCS: elective repeat cesarean section; OR: odds ratio; RR: relative risk; HR: hazard ratio; CI: confidence interval; U: Mann–Whitney U test

Study	Statistical analysis/Effect size	Results	Interpretation –Other results	Adjustment for Confounders	Results after adjusting for confounders
Chen et al. (2017) [52]	Logistic regression, Cochran-Armitage trend test. Small-to-moderate effect size	The prevalence of NDDs was significantly different between birth by CS and VD (4.7% vs. 3.8%). Children born by CS show a statistically significant association with neurodevelopmental disorders (learning difficulties, developmental delays, ADHD, sensory integration disorder, autism) (p < 0.05)	Children born by CS had 20% more possibilities to cope with NDDs in comparison with children born by vaginal delivery. Gestational age was responsible for NDDs and more specifically GA of less than 37 weeks. The increasing GA up to ≤40 to <42 weeks steadily declined the association	Child characteristics (i.e., GA, gender, birth order) and parent characteristics (i.e., mother’s age at birth, mother’s level of education, family monthly income), disease-specific factors, such as a mother's gestational weight gain and her history of gestational diabetes mellitus for children’s obesity and a parent’s history of asthma for children’s asthma	After adjusting for children’s and parents’ characteristics, the odds ratio and 95% CI for cesarean deliveries were 1.22 and 1.05–1.42, respectively. After adjusting for GA as well as for other children’s and parental variables, the link between NDDs and CS was minimized (OR =1.15; 95% CI = 0.98–1.34). Significantly more NDDs were found in children born by CS before 38 weeks. After adjusting for GA, this study finds no evidence of an important correlation between CS and NDDs in children
Chen et al. (2021) [53]	Logistic regression, Cochran-Armitage trend test. Small effect size	The NDDs rates at 8 years old were different between birth by CS and VD (5.6% vs. 5.0%). CS vs VD: OR = 1.15 (95% CI = 1.00–1.32) (p < 0.05). Significantly higher occurrence of NDDs (Learning disabilities, Developmental delay, ADHD, Sensory integration disorder, autism) was observed in children born via emergency CD than in children born via VD (OR = 1.38; 95% CI = 1.16–1.65; p < 0.05)	Different modes of delivery could influence the appearance of NDDs in childhood. CD-born children had a greater risk of NDDs than vaginally delivered children at the age of 8. Compared to children born by VD, emergency CD considerably increased the risk of NDDs by 38%. Birth < 37 or = 37 weeks is significantly associated with higher risk of NDDs (OR = 1.49; 95% CI = 1.20–1.85; OR = 1.29; 95% CI = 1.07–1.56)	Gestational age, children characteristics, socioeconomic status, maternal age at birth, maternal diseases at pregnancy	After adjusting for the previous covariates, the association between emergency CD and the occurrence of NDDs was attenuated. Emergency CS–NDDs association weakens after controlling confounders (OR = 1.27; 95% CI = 1.06–1.53). Emergency CS–NDDs association further weakens after GA adjustment (OR = 1.22; 95% CI = 1.01–1.47). These results did not support a strong association between CD and poor neurodevelopmental outcomes in children
Zhang et al. (2021) [54]	Cox proportional hazards regression analyses. Small-to-moderate effect size	Children born via planned CD have a greater likelihood to develop any NDD (HR = 1.17; 95% CI = 1.13–1.22). Additionally, planned CD was linked to ADHD (HR = 1.17; 95% CI = 1.12–1.23), ASD (HR = 1.20; 95% CI = 1.10–1.31), intellectual disability (HR = 1.26; 95% CI = 1.14–1.39), communication disorders (HR = 1.14; 95% CI = 1.02–1.28), and learning disorders (HR = 1.15; 95% CI = 1.01–1.30)	When compared to vaginal births, children born via planned or intrapartum CD had 10% to 30% higher odds of being diagnosed with any NDD, ADHD, or intellectual disability. Children born via planned CD have an elevated risk of ASD, communication problems, and learning disorders	(1) Maternal characteristics and pregnancy complications, including age at delivery, parity, highest education level at delivery, smoking during pregnancy, hypertension, diabetes, infections during pregnancy, polyhydramnios, oligohydramnios, preeclampsia or eclampsia, pelvic disproportion, placental disorders, and psychiatric history. (2) Paternal age at delivery and paternal psychiatric history. (3) Birth complications (failed induction, fetal distress, dystocia, and labor malpresentation). (4) Neonatal characteristics (birth year, sex, gestational age, small or large for gestational age birth)	1) After controlling for confounders some of the results remain significant. (2) However, after controlling for familial variables, the observed associations were attenuated to the null. The results do not suggest a causal association between CD and neurodevelopmental disorders (including learning disorders)
Bandla et al. (2017) [55]	ANOVA. Effect size not calculable	CS (SLD special education group 63.4% and SLD schools group 31.3%) is found to be related to SLD in children (p = 0.02)	Prematurity, CS, delayed speech, and family history of SLD were all significantly associated with SLD. A risk factor seems to be CS	-	-
Görker et al. (2017) [56]	Logistic regression. Large effect size	Children born via CS have a 5.675 times increased risk of having mathematic disorder (p = 0.009)	CS, developmental delays in walking, and a history of neonatal jaundice were all linked to mathematic disorder. Difficulties in mathematics are 5.675 times more likely to affect cesarean born children than vaginal born kids. A substantial risk factor for developing SLD and subtypes was a parent’s history of learning difficulties	Potential confounders were not explicitly stated in this study	-
Eaton et al. (2001) [57]	Relative risks. Moderate effect size	Children born by cesarean delivery appeared to have 50% more possibilities to cope with learning disorders (CS RR vs. VD RR; 1.7 vs. 1.38) and 25% more possibilities for “other” disorders, with both these effects being significant	Learning disorders are connected to low birth weight, small gestational age and mode of delivery. A higher risk of the less pervasive learning disorders and the “other” disorder category is found to be related to cesarean delivery	Only adjusted for gender, year of birth/other adjustments do not include mode of birth	The results remain the same after controlling for the previous confounders
Black et al. (2016) [58]	Cox proportional hazards survival analysis, binary logistic regression. Moderate effect size	Learning disability was associated with unscheduled repeat CS when compared with VBAC (3.7% vs. 2.3%; OR =1.65; 95% CI = 1.19–2.31). Learning disability risk was 65% higher after unscheduled repeat CS vs. VBAC, with no significant difference for planned CS	When compared to VBAC, children born via unplanned repeat CS had a higher prevalence of learning disabilities. Although the possibility of confounding by indication cannot be neglected, the increased risk of learning disability following unscheduled repeat CS may show the risk associated with labor in the context of a prior cesarean scar	Maternal age, gestation at birth, maternal Carstairs decile, maternal smoking status, birthweight, year of delivery, male infant, and breastfeeding at 6 weeks	The association between learning disability and unscheduled repeat CS remain the same after controlling for confounders (aOR = 1.64; 95% CI = 1.17–2.29)
Fitzpatrick et al. (2021) [59]	Logistic regression, Poisson regression. Small effect size	Children born after planned VBAC compared with ERCS had a similar risk of having a record of learning disability (OR = 1.02; 95% CI = 0.91–1.14; p = 0.754), dyslexia (OR = 1.02; 95% CI = 0.89–1.17; p = 0.765) or other specific or moderate learning difficulty (OR = 1.02; 95% CI = 0.95–1.09; p = 0.658). No significant associations found in ERCS, and planned VBAC with or without labour induction	Little evidence is provided in terms of the relation between planned mode of birth after previous cesarean and SENs in childhood. There was no significant association between learning disabilities and elective repeat CS when compared with planned VBAC	Base model - year of birth Model A - year of birth, sociodemographic (maternal age, mother’s country of birth, marital status, socioeconomic status and child’s ethnicity) and maternal medical and pregnancy- related factors (number of previous CSs, any prior vaginal birth, inter-pregnancy interval, maternal smoking status at booking, maternal BMI at booking, hypertensive disorder, diabetes and prelabour rupture of membranes). Model B - variables in model A and infant-related factors (sex of infant, gestational age at birth and birthweight centile). Model C - variables in model B and for any breastfeeding at 6–8 weeks	In the fully adjusted models, only a slight increase in the risk of any SENs (RR = 1.06, 95% CI = 1.01–1.12), other specific or moderate learning difficulties (OR = 1.10, 95% CI = 1.00–1.20) and a language or speech disorder (OR = 1.14, 95% CI = 1.00–1.30) was evident with actual VBAC compared with ERCS
González- Valenzuela et al. (2021) [60]	Binary logistic regression. Small-to-moderate effect size	Statistically significant and moderate differences were found for reading accuracy (U = 1119.50, z = −2.99, p < 0.01, r = 0.26) between the mean rank of children born vaginally (MR = 69.17) and those born by CS (MR = 48.49), for phonetic orthography (U = 1025.00, z = −3.50, p < 0.001, r = 0.31) between the mean ranks of the groups made up of the variable type of delivery, (MR = 70.30) vs. (MR = 46.13) and for visual orthography (U = 1262.00, z = −2.24, p < 0.05, r = 0.20), (MR = 67.48) vs. (MR = 52.05). Higher odds of phonetic orthography (42.5% vs. 17.9%; OR = 3.40; 95% CI = 1.47–7.87), visual orthography (40.0% vs. 17.9%; OR = 3.06; 95% CI = 1.32–7.13), and reading accuracy difficulties (42.5% vs. 16.7%; OR = 3.69; 95% CI = 1.58–8.64) in CS vs VD	The mean values for children born vaginally show noticeably higher scores in the learning variables. Cesarean birth is a potential independent risk factor for problems with phonetic and visual orthography, as well as reading accuracy	Gestational, obstetric, and neonatal variables (maternal age at delivery, gestational age, fetal presentation, Apgar 1, and newborn weight)	For the main effect between delivery mode and phonetic orthography, fetal presentation appears to be a potential modifying and confounding factor (aOR = 2.50; 95% CI = 1.01–6.18). All the other results remain the same
Zou et al. (2023) [61]	Multivariate logistic regression, LASSO regression model. Moderate-large effect size	No statistically significant differences were found between DD and delivery mode. DD was associated with lower maternal education levels and parenting styles (OR = 4.93, 95% CI = 1.11–21.91), mothers malnutrition during pregnancy (OR = 10.31, 95% CI = 1.84–37.86), children’s daily exposure to second-hand smoking (OR = 5.33, 95% CI = 1.52–18.66)	DD may be influenced by the home reading environment, and various educational, sociometric, and demographic factors. In this sample, association was also found between dyslexia and birth weight (p = 0.006), as well as feeding patterns. (p = 0.049). No association was found between the mode of delivery and DD (p = 0.147)	In this study, adjustments for confounders were made only in the cases of associations between Chinese Dyslexia and the Home Reading Environment	-
